# The surprising complexity and diversity of sperm storage structures across Galliformes

**DOI:** 10.1002/ece3.11585

**Published:** 2024-06-21

**Authors:** Katherine Assersohn, J. Paul Richards, Nicola Hemmings

**Affiliations:** ^1^ School of Biosciences University of Sheffield Sheffield UK

**Keywords:** cryptic female choice, fertility, post‐copulatory sexual selection, reproduction, sperm selection, uterovaginal junction

## Abstract

In internal fertilisers, the precise timing of ovulation with the arrival of sperm at the site of fertilisation is essential for fertilisation success. In birds, mating is often not synchronised with ovulation, but instead females utilise specialised sperm storage tubules (SSTs) in the reproductive tract, which can ensure sperm are always available for fertilisation at the time of ovulation, whilst simultaneously providing a mechanism of post‐copulatory sexual selection. Despite the clear importance of SSTs for fertilisation success, we know little about the mechanisms involved in sperm acceptance, storage, and release. Furthermore, most research has been conducted on only a small number of species, based on which SSTs are usually assumed to look and function in the same way across all species. Here, we conduct a comparative exploration of SST morphology across 26 species of Galliformes. We show that SSTs, and the surrounding tissue, can vary significantly in morphology across species. We provide observational evidence that Galliformes exhibit at least 5 distinct categories of tubule types, including distinctive coiled and multi‐branched tubules, and describe 2 additional features of the surrounding tissue. We suggest functional explanations for variation in tubule morphology and propose next steps for future research. Our findings indicate that SSTs are likely to be far more variable than has previously been assumed, with potentially important consequences for our understanding of sperm storage in birds and post‐copulatory sexual selection in general.

## Graphical abstract

Female birds utilise specialised sperm storage tubules in the reproductive tract, which ensure sperm are always available for fertilisation, whilst also potentially facilitating female sperm selection . Here, we show that sperm storage tubules, and the surrounding tissue, can vary substantially in morphology across species of Galliformes. We present 5 distinct categories of tubule and describe 2 additional features of the surrounding tissue. These findings have important implications for our understanding of sperm storage in birds and post‐copulatory sexual selection in general.

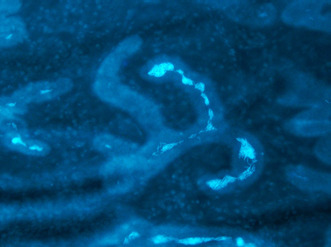

## 1 INTRODUCTION

In internal fertilisers, successful fertilisation relies on the arrival of sperm at the site of fertilisation at the precise time of ovulation. In many species, ensuring sufficient sperm are available for fertilisation requires insemination to be precisely timed with the release of the ovum. In others, insemination and ovulation are not synchronised, sometimes occurring many days or even months apart (Birkhead, 1992; Birkhead & del Nevo, 1987; Hatch, 1983; Hemmings & Birkhead, 2020; Wanless & Harris, 1986). To compensate for this, females may store sperm within the reproductive tract, maintaining it in a viable state to be released at ovulation (Bakst, 2011).

Female sperm storage is a widespread phenomenon, occurring in many invertebrates and all major vertebrate groups (Holt & Fazeli, 2016; Shankar et al., 2022). In species exhibiting sperm storage (as opposed to just sperm longevity), females can store sperm for a few hours to months or even years (Holt & Fazeli, 2016; Levine et al., 2021; Orr & Zuk, 2012). This is achieved through providing an environment favourable for sperm survival, often in the form of specialised morphological structures e.g., seminal receptacles, (gastropods and arthropods), spermathecae (many invertebrates), tubules (birds, reptiles, fish and amphibians), or a combination of both spermathecae and seminal receptacles (*Drosophila melanogaster*) (reviewed in; Holt, 2011 and Orr & Brennan, 2015). Sperm storage structures not only guard against a lack of sperm at fertilisation but may also allow females to preferentially store/release sperm from preferred males, thereby providing a mechanism of postcopulatory sexual selection (i.e., cryptic female choice) (Bakst, 2011; Eberhard, 1996; Mendonca et al., 2019; Sasanami, 2017).

Sperm storage has been particularly well‐studied in birds, beginning with speculation in the early‐1900s that viable sperm were capable of surviving in the oviduct long after insemination (Payne, 1914). It was not until the mid‐1900s that the first specialised storage sites, then termed ‘sperm nests’, were identified and suggested to be responsible for sustained fertility in female birds (Van Drimmelen, 1946). ‘Sperm nests’ were initially described as being located within shallow crypts of the infundibulum (the site of fertilisation), but in a series of histological studies, sperm storage was later suggested to occur principally at the utero‐vaginal junction (UVJ), a small non‐distinct section of the vagina bordering the uterus (Bobr, Lorenz, & Ogasawara, 1964; Bobr, Ogasawara, & Lorenz, 1964; Verma & Cherms, 1964, 1965). Today, these structures are commonly known as ‘sperm storage tubules’ (SSTs), occurring primarily in the UVJ, with evidence for infundibular storage sites remaining equivocal (but still widely cited) (Assersohn et al., 2021; Bakst, 2011; Sasanami, 2017).

Although commonly defined as ‘simple tubular invaginations’, SSTs are increasingly appreciated as being highly specialised, with an expanding body of evidence to suggest that the molecular and biochemical processes occurring within SSTs are complex and highly regulated (Bakst, 2011; Freedman et al., 2001; Hemmings et al., 2015; Holm et al., 2000; Khillare et al., 2018; Mendonca et al., 2019; Sasanami, 2017). The vagina is typically hostile to sperm: only 1% of inseminated sperm make it into storage (Bakst, 2011), and it is thought to be a selective process that probably ensures sperm with atypical morphology and physiology are inhibited from participating in fertilisation (Bakst, 1994; Bobr, Lorenz, & Ogasawara, 1964; Khillare et al., 2018; Ogasawara et al., 1966). Once accepted into SSTs, numerous compounds are produced that likely act to suppress sperm motility and provide protection from structural damage and oxidative stress (Bakst & Bauchan, 2015; Freedman et al., 2001; Holm et al., 2000; Huang et al., 2016, 2017; Khillare et al., 2018; Matsuzaki et al., 2015; Mendonca et al., 2019; Sasanami, 2017). At ovulation, sperm are re‐mobilised and released from storage (Hiyama et al., 2014; Ito et al., 2011). In birds, there is a very narrow window of time (around 15 min in domestic fowl; Bakst, 1994) between ovum release and the laying down of the outer perivitelline layer (a matrix of glycoproteins that surrounds the ovum and blocks further penetration by sperm; Hemmings & Birkhead, 2015). The timing of acceptance and release of sperm is therefore highly regulated, probably through fine hormonal and possibly nervous control (Hemmings et al., 2015). Furthermore, 3D imaging of SSTs in zebra finches (*Taeniopygia guttata*) has identified gate‐like constricted openings that may act to limit the ability of sperm swimming below a certain velocity to enter storage, providing an additional mechanism of selection for high‐quality sperm (Hiyama et al., 2014; Ito et al., 2011; Mendonca et al., 2019). Despite these advances, we still lack a comprehensive understanding of the mechanisms by which SSTs accept, store/maintain and release sperm (Khillare et al., 2018; Sasanami, 2017; Shankar et al., 2022). We have long known that even among commercial birds selected for consistent and high fertility, sperm storage duration varies greatly. For example, sperm are stored for just 5–10 days in Japanese quail (*Coturnix japonica*) but up to 15 weeks in turkeys (*Meleagris gallopavo*) (Birkhead & Møller, 1990; Sasanami, 2017). However, the causes of such variation are not known or generally discussed in the context of SST diversity across avian species.

Perhaps an even more fundamental barrier to our understanding of sperm storage in birds is our lack of knowledge of inter‐specific variation in SST morphology. Sperm are some of the most diverse cells in the animal kingdom (Pitnick et al., 2009), and so it stands to reason that the structures that selectively store them might also vary considerably in both structure and function (Cramer et al., 2023). Sperm length has been found to correlate negatively with SST number and positively with SST length in passerines (Briskie & Montgomerie, 1993), suggesting that the co‐evolutionary dynamics between male and female post‐copulatory sexual selection may be a driving factor in the evolution of sperm morphology (Kustra & Alonzo, 2023). Sperm morphology and female sperm storage organ morphology have been shown to correlate in other taxa as well (mainly invertebrates; Higginson et al., 2012; Miller & Pitnick, 2002). Within‐population variation in sperm storage organ morphology and function has also been documented in *Drosophila* (Lüpold et al., 2013). However, the general assumption in birds is that SSTs always look – and function in – the same way as those observed in the species in which SSTs have been most well studied (e.g., domestic chickens (*Gallus gallus domesticus*), turkey, Japanese quail, and the zebra finch). In studies that have identified SSTs, there is also inconsistency in how quantitative variables such as SST number and length are measured, making cross study comparison difficult. For example, there is no consensus on whether branched tubules should be considered (and measured as) one tubule with great total length, or many distinct shorter tubules. An important step in determining the degree of variation in SST form and function across species, and how this correlates with post‐copulatory processes, will be to develop more consistent and reproducible descriptions and measures of SST traits.

Here, we report and present the discovery of remarkable variation in morphological structure of SSTs across species of Galliformes. We also present methods for dissecting and examining the folds of the utero‐vaginal junction in birds, suggest criteria for categorising tubule morphology based on our observations, and discuss the potential for future research in Galliformes and across birds in general.

## 2 MATERIALS AND METHODS

### 2.1 Animals

Galliformes are a very large, diverse, and well‐studied bird group in which body size and mating system vary greatly, and samples are easily accessible, making them ideal for exploring interspecific variation in SST morphology. We collected the whole oviduct of a single female from 28 different species of Galliformes. Dead birds were sourced in 2016 from breeders in the process of disposing of excess stock, and dissections took place on site within 30 min of each bird being killed. Females were included in the study only if they were in breeding condition (confirmed by the presence of an ovum in the oviduct and/or a hierarchy of developed ova in the ovary), since the avian oviduct is known to regress in size outside of the reproductive period. Males and females were housed together, allowed free access to each other, and all copulations were natural. Females were dissected at most within 1–2 days of commencing egg laying, and all females were confirmed to have laid fertile eggs (demonstrating that they had recently accepted and stored sperm).

**FIGURE 1:**
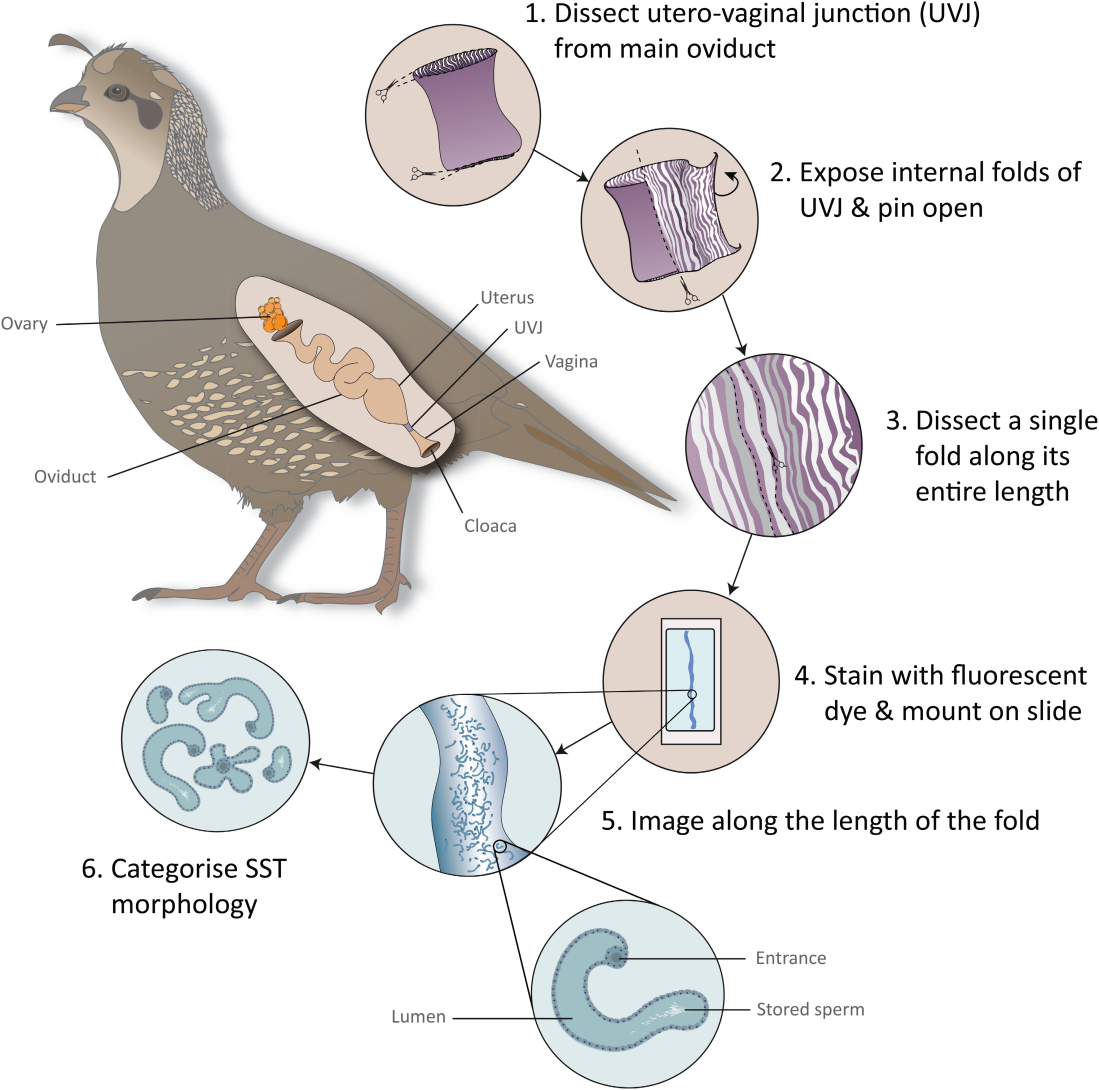
Schematic illustrating the avian oviduct and ovary, and the methods of UVJ dissection, fold dissection and SST categorisation. Within the oviduct, the utero‐vaginal junction (UVJ) (purple segment) – the site of primary sperm storage, was dissected and pinned open to expose the internal folds. A single fold was then dissected along its length and stained with Hoechst fluorescent dye. The fold was then laid flat and fixed onto a slide before being imaged along its entire length using fluorescent microscopy. SSTs are typically visible along the centre of UVJ folds. Schematic drawn in Adobe Illustrator (V.28.4.1).

The oviduct was removed intact (including the cloaca and ovary), unravelled, stripped of connective tissue, and briefly cleaned in phosphate‐buffered saline (PBS). The oviduct was then pinned out lengthways in a long, shallow wax‐based tray, where it was photographed and measured. The length of each individual section of the oviduct, as well as its entire length, was measured. We also recorded the wet mass of the oviduct after briefly dabbing off excess liquid with absorbent tissue. Once all measurements were complete, the oviduct was transferred into a deeper tray, pinned so that each section was straight, and submerged in 10% formalin solution to fix the tissue. After at least 48 h in fixative, a segment of the vagina containing the UVJ was then cut away from the rest of the oviduct (Figure 1; step 1; Briskie & Birkhead, 1993). Since the UVJ has previously been reported to be located at the uterus end of the vagina, the segment was always cut at the beginning of the uterus. However, due to the use of vaginal tissue for another study, the degree to which the segment extended along the vagina varied from 19 to 30% of the total vaginal length. Upon microscopic examination of each sample, the start of the UVJ segment was designated as the point at which SST first appeared, and the end of the UVJ segment was where uterine tissue started. Occasionally, it appeared that the distribution of tubules continued slightly further down the vagina than the length of our sample allowed us to visualise; however, this was uncommon (only occurring in 4 species; California quail (*Callipepla californica*), European quail (*Coturnix coturnix*), mountain quail (*Oreortyx pictus*), and Temminck's tragopan (*Tragopan temminckii*)), and the dissected segments were expected to contain the majority (if not all) of the SSTs in the sample.

### 2.2 Dissection

Samples were cut longitudinally with micro‐dissecting scissors, pinned open with butterfly specimen pins on Sylgard™ 184 gel (Sigma‐Aldrich), and covered with phosphate buffered saline (PBS), to reveal the internal luminal mucosa and mucosal folds of the vagina (Figure 1; step 2). For each sample, 2 folds were dissected. Folds were examined under a Leica MZ75 dissecting microscope and dissected by cutting the entire length of one side of the ‘valley’ (as opposed to the crest) of a single fold along the entire length of the sample (Figure 1; step 3) (Briskie & Birkhead, 1993). Care was taken to remove only the mucosa, peeling it away from the underlying connective tissue (the lamina propria), but avoiding cuts to the underlying tissue. Before cutting along the remaining side, the fold was gently prised open along the length. Opening the fold out whilst it was still attached to the sample along one side had the benefit of providing leverage for gently teasing apart the ‘crest’ of the fold. Flattening the crest was necessary to allow the fold to lie smoothly once mounted, but was much more difficult to achieve once the fold was fully dissected. If necessary, additional cuts were made to remove connecting tissue whilst ensuring the integrity of the fold was maintained and the SSTs were not damaged. The entire length of the remaining attached side was then cut longitudinally, and the fold gently removed from the underlying tissue. The fold was then incubated for 5–10 min with 10–20 μL of Hoechst 33342 dye (the exact volume depended on how much was needed to fully submerge the fold). Hoechst was used to help visualise sperm in storage, which aided in identifying SSTs, and had the added benefit of clarifying the edges of the SSTs against the surrounding tissue. The fold was then placed onto a slide (lamina propria side down) and gently opened to lie flat. Once flattened along the entire length, a coverslip was added with Fluoromount‐G™ Aqueous Mounting Medium (Sigma‐Aldrich) and left to dry. Dried samples were sealed with transparent nail varnish and kept in the dark until imaging (Figure 1; step 4).

### 2.3 Morphological observations from dissection

We observed a remarkable amount of variation in the thickness, texture, integrity, and overall appearance of vaginal tissue between species. The visual presentation of the folds of the vagina varied between species, with some species having large and very distinct folds whilst in others the individual folds were less pronounced. Some species displayed obvious pigmentation in the vagina, and in many cases, tubules were visible by eye at this stage (Figure 1; step 5). There was always an obvious difference between vaginal and uterine tissue, though in some species this difference was more pronounced than others. Uterine tissue was always to some degree thicker, darker in appearance and with larger/taller folds relative to the vagina.

Samples also clearly varied in their response to preservation in formalin: While some samples remained very well preserved, others had become fragile and difficult to dissect. In the case of 2 species, Javan peahen (*Pavo muticus*), and Grey partridge (*Perdix perdix*), samples were so fragile they were impossible to dissect. Consequently, we removed these species from the sample pool, reducing the sample size to 26 species (52 folds). This difference in response to preservation, ease of dissection and mounting suggests there are some innate differences in tissue structure between species; however, most samples remained in suitable condition for dissection, despite there being an approximate 7‐year gap between tissue fixation and sample dissection.

### 2.4 Imaging

Slides were examined under fluorescent and brightfield light on a Leica DMLB compound microscope with an LEJ ebq100 fluorescence source. Images were captured with Infinity Analyse software via a Lumenera Infinity3 USB camera. Images were taken along the entire length of each fold, following the centre line of the fold (Figure 1; step 5), at 50× magnification, with one image directly bordering the next with no overlap and no missing sections. Depending on the individual sample, images were taken either using brightfield, darkfield, or fluorescent illumination, or a combination, in whichever way produced the highest quality image for that sample. In many cases, images of one field of view were taken repeatedly through multiple planes to avoid missing hidden structures where possible. Image scale bars were produced in ImageJ (Schneider et al., 2012), and contrast and brightness were adjusted in Adobe Photoshop (version 25.5) to aid visualisation where necessary. For some structures of note, additional images were taken at either 100 or 200× magnification.

### 2.5 Tubule categorisation

On microscopic examination of each image, careful notes of visible structures were made that were subsequently assessed to create distinct categories that could be used to define consistent features (Figure 1; step 6). An initial criterion for categorising structure type was then created, which was used to categorise the visible structures within each image across all samples. A second observer then used the categorisation criteria to categorise a subset of images: one image per sample (two samples per species; 52 images in total) was chosen for re‐categorisation, at random (using a random number generator), from a pool of images that only contained visible structures (i.e., excluding images in which no features were observed). Repeatability of categorisation was then calculated in R (R Core Team, 2023) using the package rptR (Stoffel et al., 2017). A binary family was used for each model, with tubule category as the response variable and sample ID as the random effect. For categories with lower repeatability, adjustments to the categorisation criteria were made to improve reproducibility. We then used these revised categorisation criteria to create a flow chart (also reproduced as a dichotomous key in Appendix S1) that could be used and built upon by future researchers to categorise the structures they observe in their own samples, with the aim of encouraging a consistent and definitive use of terminology throughout the literature. We acknowledge that a greater number of distinct structures are likely to exist across species and encourage future work to critically examine the structures observed and consider whether they fit into the existing categories presented here, or whether new categorisations are necessary.

To aid in categorising tubule location, we considered the samples as being divided into 3 sections, each containing an equal number of images: C (the most distal section, i.e., closest to the cloaca and bordering the rest of the vagina), M (the central third of the sample), and U (the most proximal section that borders the uterus; Briskie, 1996). If the sample contained an uneven number of images making equal thirds impossible, the M section was expanded to accommodate the additional images, with the C and U sections always containing the same number of images and so being proportionally the same size. Note that we always consider the first image in C to be the start of the UVJ region of the vagina and will contain the first appearance of tubules.

## 3 RESULTS

### 3.1 Tubule location within the UVJ

Tubule location varied between species; however, in the majority of species (20; 76.9% of species), tubules were found in all three regions of the sample (C, M, and U) in at least one of the sample folds. In 13 species (50% of species), tubules were only found in the C and M (but not the U) regions in at least one of the sample folds, but of these, 6 species never had tubules in the U region of either fold: harlequin quail (*Coturnix delegorguei*), Japanese quail, mountain quail, Swinhoe's pheasant (*Lophura swinhoii*), silver pheasant (*Lophura nycthemera*), and wild turkey. There was only 1 species (*California quail*) in which tubules were seen only in the C region (and not the M and U), but this was only the case for one fold of the sample. Taken together, tubules were commonly found across the entire length of the sample, but in half of all species, at least one fold sample did not have tubules that directly bordered the uterus.

### 3.2 Presentation of fold tissue

Variation in the texture of vaginal and uterine tissue between species was apparent upon microscopic examination: In some cases, tissue was thin, and tubules were clear, and in others, the tissue was thick and layered, with tubules sometimes being embedded making them more difficult to identify, even when examining through multiple planes of focus. Sperm was not always observed in storage in every sample, possibly due to lower retention of sperm, fewer copulations, or fewer sperm being transferred during copulation to begin with. In samples that contained stored sperm, the distribution of sperm was uneven throughout the samples, with most tubules being free of sperm.

### 3.3 Channels

**FIGURE 2:**
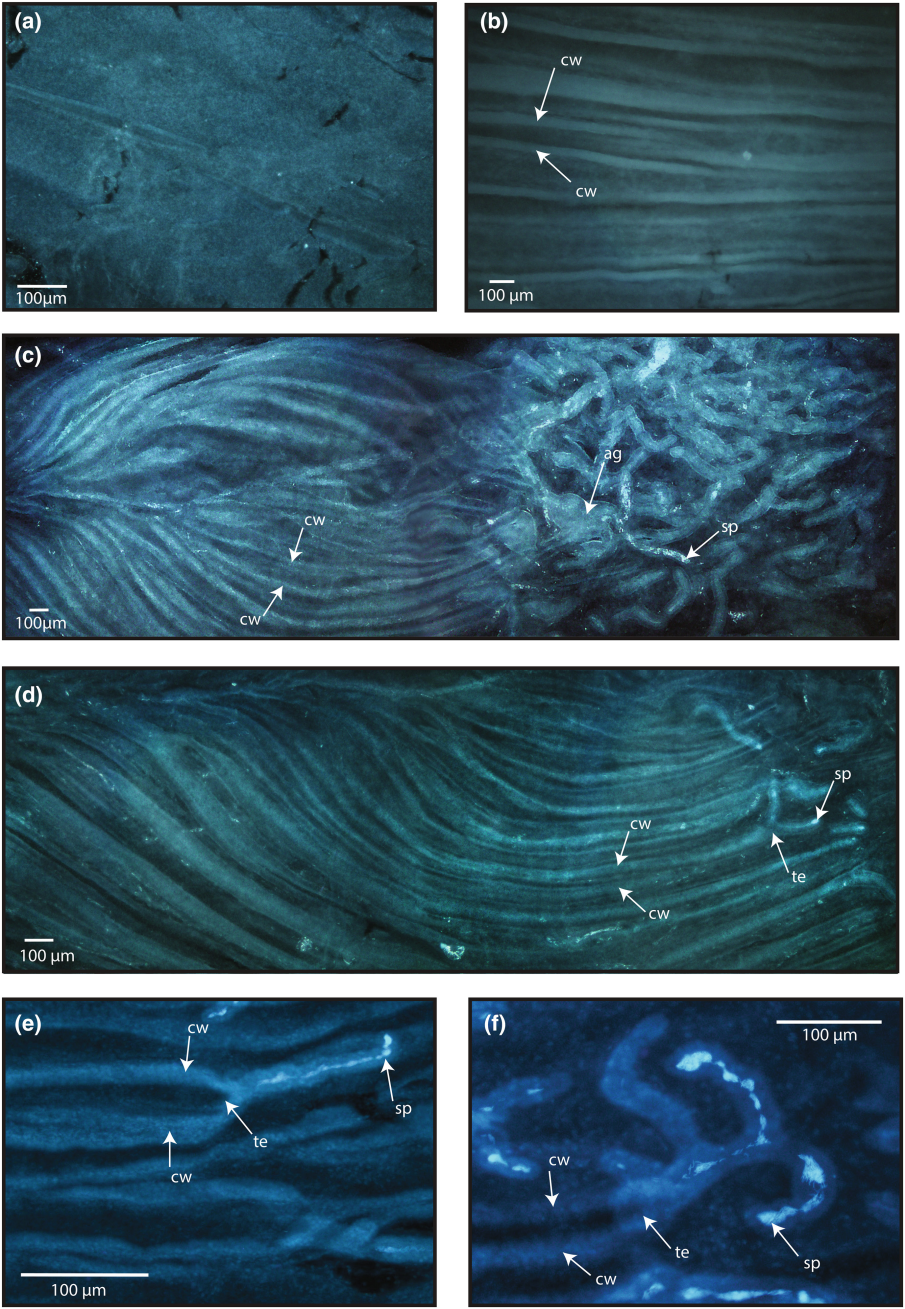
(a) An example of the typical presentation of UVJ tissue that does not contain channels or SSTs, from European quail (*Coturnix coturnix*) at 50× magnification. (b) Channel‐like structures of the UVJ tissue from helmeted guineafowl (*Numidea meleagris*) at 50× magnification. (c and d) Channel‐like structures of the UVJ tissue in grey francolin (*Francolinus pondicerianus*) at 50× magnification. Each image crosses 3 fields of view. Channels are very long and end in a region populated by tubules. In (c), you can see a large globular (agglomerate) branched tubule (ag) and channels that appear to end directly in a tubule with obvious stored sperm (sp). (d) Particularly clear example of a long channel ending in a forked tubule. (e) Slightly different presentation of channel tissue in mountain quail (*Oreortyx pictus*) at 100× magnification. Channels are shorter in length, funnel‐like and end directly in tubules. (f) Example of channels apparently ending in a highly branched tubule in mountain quail at 200× magnification. Stored sperm is visible in more than one branch. Arrows point to representative (but not all) examples of channel walls (cw), tubule entrances (te), stored sperm (lighter/brighter blue) (sp), and agglomerate tubules (ag) (where relevant). Images are all orientated with the cloaca closest to the left side and the uterus closest to the right.

We repeatedly observed ‘channel‐like’ structures (here‐on referred to as channels) that presented as grooves along a single fold and often extended along a large portion of the fold (see Figure 2a where no channels are present, relative to Figure 2b–f where channels can be seen). Although occasionally shorter and more ‘funnel‐like’ in appearance (Figure 2e,f, seen in; black francolin (*Francolinus francolinus*), grey junglefowl (*Gallus sonneratii*), golden pheasant (*Chrysolophus pictus*), Lady Amherst's pheasant (*Chrysolophus amherstiae*), mountain quail, red legged partridge (*Alectoris rufa*), roul roul partridge (*Rollulus rouloul*), and Temminck's tragopan), these structures were distinct in presentation from tubules but contained clear thickened tissue on either side (channel walls) with a ‘lumen‐like’ depression along the centre. Channels were sometimes seen beginning at the start of the sample (the C region) and ending at the presence of tubules (Figure 2c), and they either extended strictly in parallel or merge and split into a series of interconnected pathways. They often (but not always) traversed more than one field of view (Figure 2c,d). In some cases, channels appeared further along the sample and were occasionally seen to end directly in a tubule (Figure 2c–f). Whilst in many cases channels appear as ‘open‐top’ grooves (e.g., Figure 2b,e,f), it was not always clear from 2D imaging whether in some cases they may be enclosed tubes (e.g., Figure 2c,d). Ultimately, future work exploring the 3D ultrastructure of channels will be needed to determine this.

**FIGURE 3:**
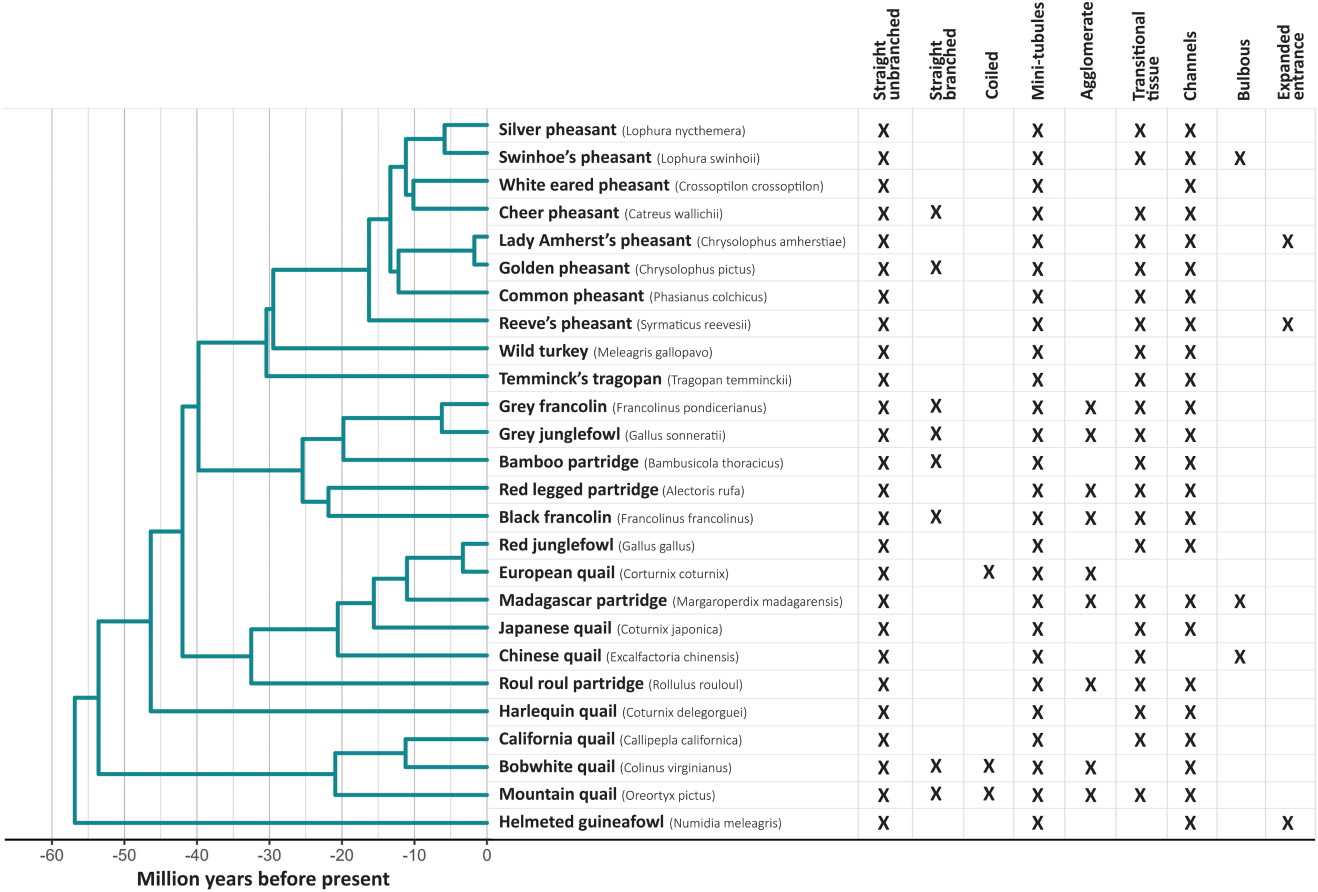
Phylogeny for the 26 species examined, and the tubule and tissue types observed within those species. Cells marked with ‘X’ indicate that this species exhibited this tubule or tissue type to some degree in at least one fold of the sample. Galliformes phylogeny with time‐calibrated branch lengths was obtained from Stein et al. (2015), which was trimmed using the R package treeplyr (Harmon, 2023).

Channels were observed to some degree in most species examined (24 species (92.3% of all species) and 46 folds (88.5% of fold samples); Figure 3). There were only 2 species where channels were observed in one fold but not the other (black francolin and Madagascar partridge (*Margaroperdix madagarensis*)), and only 2 species where no channels were observed in either fold of the sample (European quail and Chinese quail (*Excalfactoria chinensis*)). The categorisation of channel tissue was significantly repeatable between observers (*R*_Linkscale_ = 0.99, CI = 0.99, 1.00, *p* < .0001).

### 3.4 Transitional tissue

**FIGURE 4:**
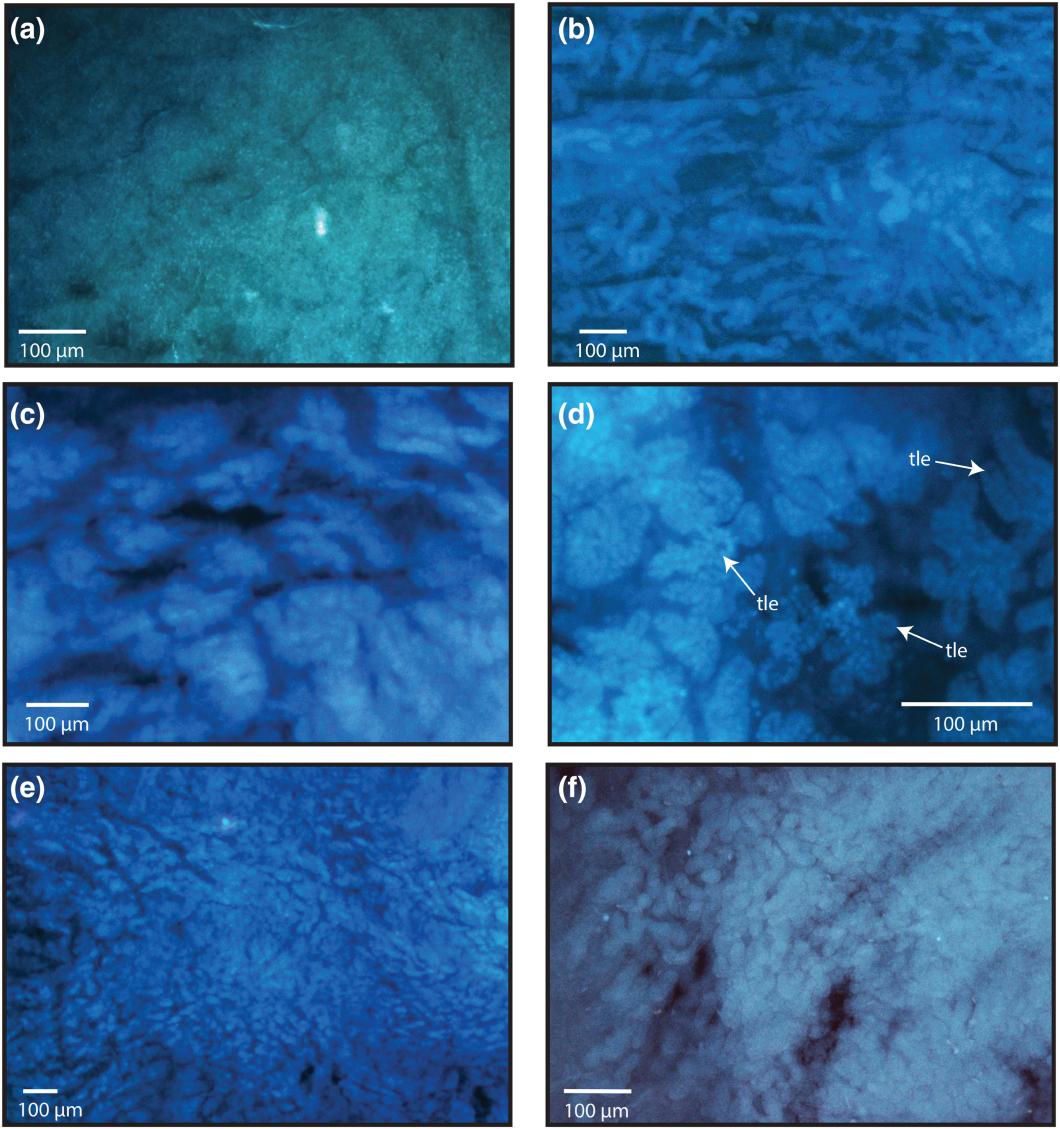
(a) Example of typical (non‐transitional) tissue at the uterus end of the vagina in black francolin (Francolinus francolinus) at 100× magnification. (b) Transitional tissue in Japanese quail (*Coturnix japonica*) at 50× magnification. Tissue appears like poorly defined tubule tissue, much smaller than tubules and in places difficult to distinguish from surrounding tissue. (c) Mountain quail (*Oreortyx pictus*) at 50× magnification. Distinct clusters of agglomerate‐like tissue that is distinct from uterine tissue but with a poorly defined ‘fluffy’ appearance. When viewed under high magnification, looks like smaller less well‐defined agglomerate tissue (see d for an example). (d) Transitional tissue from Mountain quail at 200× magnification. (e) Transitional tissue from Harlequin quail (*Coturnix delegorguei*) at 50× magnification. Tissue appears like extremely densely packed tiny tubules, distinct from storage tubules in being overall significantly smaller, but with tubule like entrances only seen when examined under high magnification. (f) Transitional tissue in Temminck's tragopan (*Tragopan temminckii*) at 100× magnification, showing a clear transition from tubule‐like (but poorly defined) tissue in the vagina (to the left) to dense uterine tissue (on the right). Arrows point to representative (but not all) examples of tubule‐like entrances (tle). Images are all orientated with the cloaca closest to the left side and the uterus closest to the right.

Across most species, we observed structures that we could neither conclusively define as tubule tissue nor vaginal or uterine tissue. These structures always directly preceded the uterus, and often looked similar in morphology to tubules seen further down the sample, but in this case were either poorly defined, and/or much smaller and increasing in density until merging into uterine tissue. We refer to these structures as ‘transitional tissue’, as they appear transitional between regular tubules and uterine tissue. In many cases, when viewed at high magnification, very small tubule‐like entrances could be seen (see Figure 4c,d for a comparison of transitional tissue at low and high magnification). Transitional tissue was not considered to be tubule tissue as we did not deem it likely to be functioning in sperm storage.

Transitional tissue was never seen to contain stored sperm and was observed to some degree in 22 species (84.6% of species) and 40 folds (76.9% of fold samples) with only 4 species not displaying transitional tissue in either fold of the sample (bobwhite quail (*Colinus virginianus*), European quail, helmeted guineafowl (*Numidia meleagris*), and white eared pheasant (*Crossoptilon crossoptilon*)) (Figure 3). The distinction between tubules approaching the U region of the UVJ and early transitional tissue was sometimes difficult and required a degree of subjectivity; however, the categorisation of transitional tissue was significantly repeatable between observers (*R*_Linkscale_ = 0.97, CI = 0.98, 1.00, *p* < .0001).

### 3.5 Tubule categorisation

In addition to transitional tissue and channel tissue, we found five distinct categories of tubules.

**FIGURE 5:**
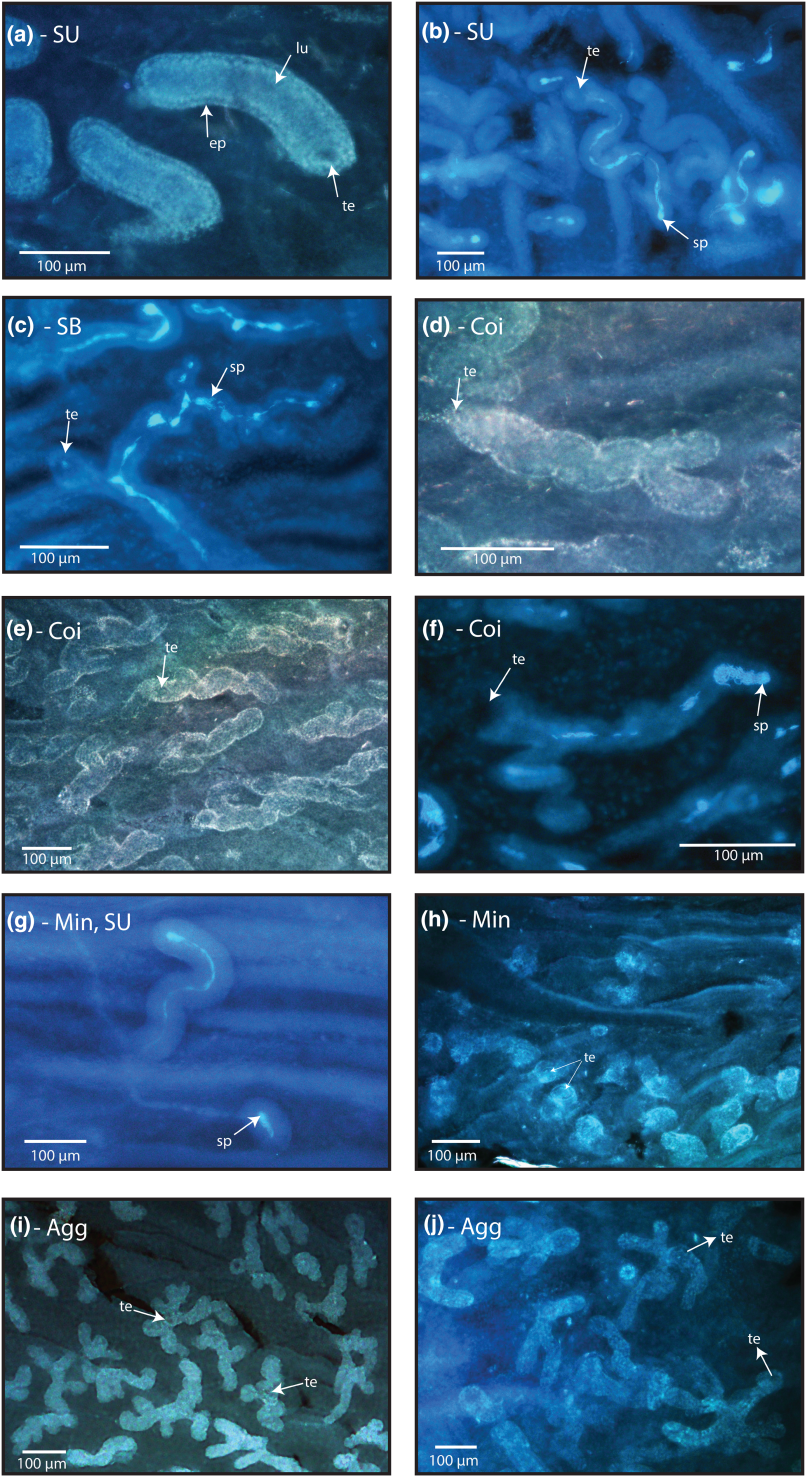
(a) Example of straight unbranched tubules in bamboo partridge (*Bambusicola thoracicus*) at 200× magnification; (b) example of straight unbranched tubules in common pheasant (*Phasianus colchicus*) at 100× magnification; (c) example of straight branched tubules in mountain quail (*Oreortyx pictus*) at 200× magnification; (d) example of a branched tubule that is also coiled, in bobwhite quail (*Colinus virginianus*) at 200× magnification. It is common for coiled tubules to also be branched; (e) An example of a large number of coiled tubules in bobwhite quail at 100× magnification; (f) An example of a branched tubule where one branch is coiled but not the other, in mountain quail at 200× magnification; (g) An example of a mini‐tubule and a regular sized tubule in the same field of view in wild turkey (*Meleagris gallopavo*) at 100× magnification. The mini‐tubule has visible stored sperm; (h) An example of many mini‐tubules in close proximity to one another in bobwhite quail at 100× magnification. Some tubules are so short in length they appear as little more than an entrance; (i) An example of agglomerate tubules in black francolin (Francolinus francolinus) at 50× magnification. Tubules are so highly branched they appear as globular rather than tubular structures; (j) An example of agglomerate tubules in grey junglefowl (*Gallus sonneratii*) at 100× magnification. Tubules are so highly branched they form some unique and interesting shapes. Arrows point to representative (but not all) examples of the tubule lumen (lu), the tubule epithelium (ep), tubule entrances (te), and stored sperm (lighter/brighter blue) (sp). Images are all orientated with the cloaca closest to the left side and the uterus closest to the right. Image title abbreviations are as follows: Straight unbranched – SU; straight branched – SB; coiled – Coi; Mini‐tubules – Min; Agglomerate tubules – Agg.

**FIGURE 6:**
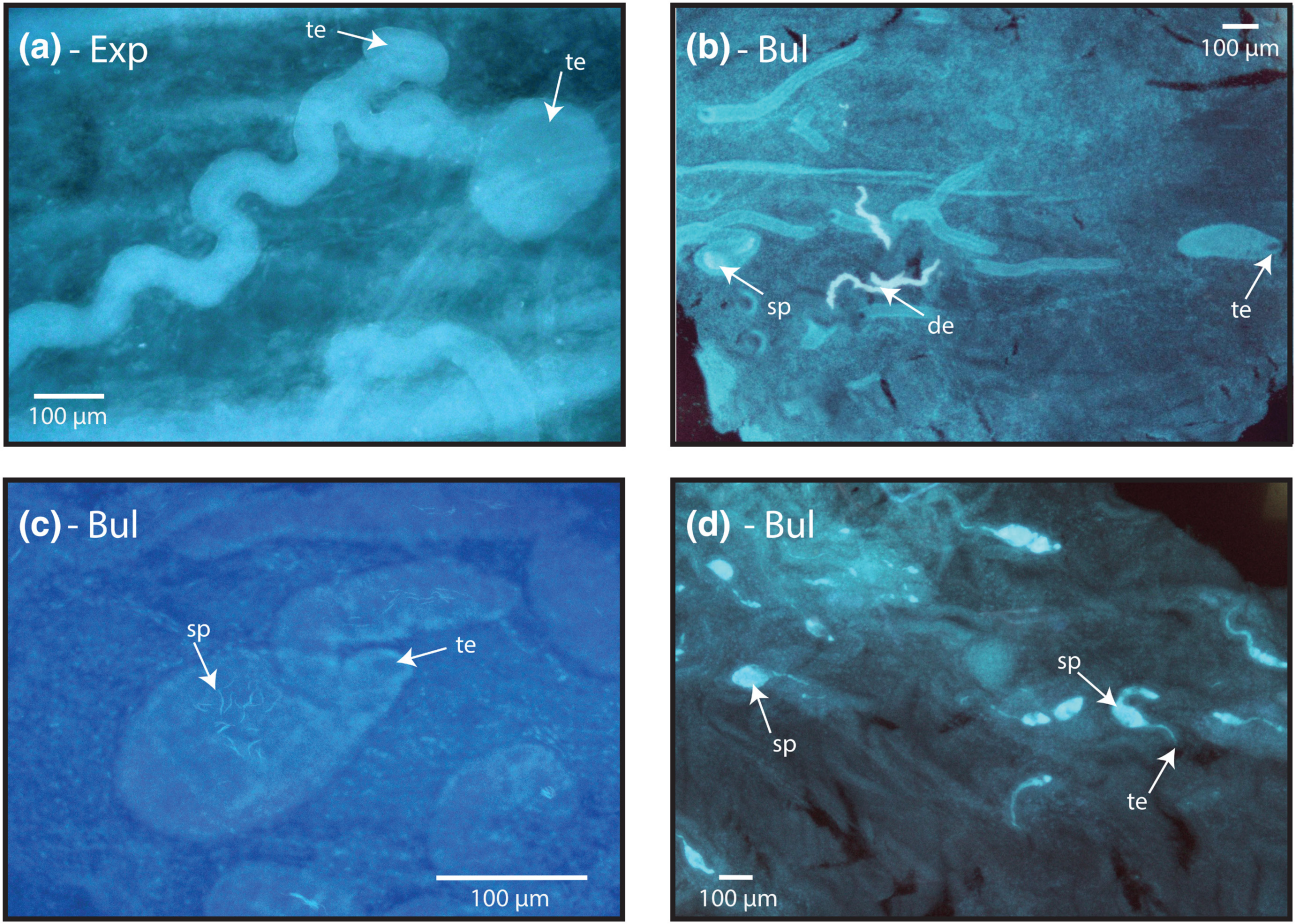
(a) Example of tubule/s with ‘expanded’ entrances, in helmeted guinea fowl (*Numidia meleagris*) at 100× magnification, with tubule/s that appear to have very wide entrances. It is not clear whether this is two overlapping tubules or a single tubule with multiple entrances. (b) Examples of ‘bulbous’ types in Chinese quail (*Excalfactoria chinensis*) at 50× magnification, with 2 tubules that appear engorged relative to the other tubules in the sample. One tubule has obvious stored sperm. (c) Examples of ‘bulbous’ types in Swinhoe's pheasant (Lophura swinhoii) at 200× magnification, with a smaller number of stored sperm. (d) Examples of ‘bulbous’ types in Madagascar partridge (*Margaroperdix madagarensis*) at 50× magnification with clear stored sperm. Arrows point to representative (but not all) examples of tubule entrances (te), stored sperm (lighter/brighter blue) (sp) and some non‐tissue debris that has picked up the dye (de). Images are all orientated with the cloaca closest to the left side and the uterus closest to the right. Image title abbreviations are as follows: Bulbous – Bul; expanded – Exp.

**FIGURE 7:**
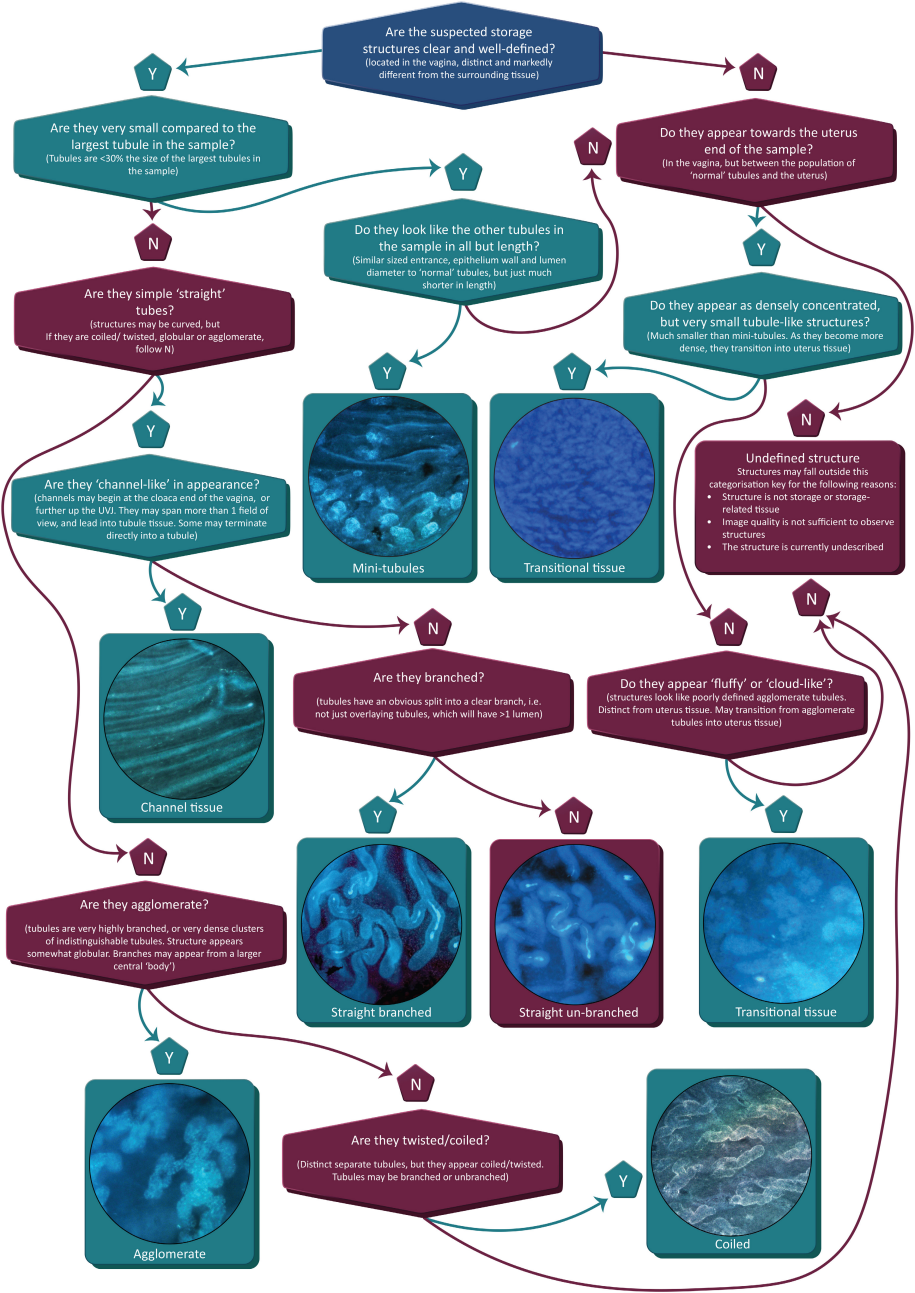
Categorisation flowchart for determining tubule type in Galliformes. Images within this flowchart have been cropped and expanded for easy visualisation, but are taken from the following species: mini‐tubules – bobwhite quail (*Colinus virginianus*) at 100× magnification; transitional tissue (top) – Harlequin quail (*Coturnix delegorguei*) at 50× magnification; channel tissue – grey francolin (*Francolinus pondicerianus*) at 100× magnification; straight branched – Reeves pheasant (*Syrmaticus reevesii*) at 100× magnification; straight unbranched – common pheasant (*Phasianus colchicus*) at 100× magnification; transitional tissue (bottom) – black francolin (*Francolinus francolinus*) at 50× magnification; agglomerate – black francolin at 100× magnification; coiled – bobwhite quail at 100× magnification. This flowchart has also been provided in a dichotomous key format in Appendix S1, which may prove useful for practical applications.

1. Straight unbranched tubules – the simplest structure, with a clear single lumen. They may bend but they do not coil or branch. At least some proportion of tubules within every sample was straight unbranched, making it the most common type of tubule (Figure 3). The categorisation of straight unbranched tubules was highly repeatable between observers (*R*_Linkscale_ = 0.80, CI = 0.40, 0.99, *p* = <.0001). Examples can be seen in Figures 2, 5, and 6.
2. Straight branched tubules – generally possessing between 1 and 3 long branches (though occasionally more), without coils or twists. As with straight unbranched tubules, branches may bend. These are commonly described in the literature and were present to some degree in 8 species (30.8% of species). For each species that contained straight branched tubules, they were common and found in both folds of the sample (Figure 3). The categorisation of straight branched tubules was highly repeatable between observers (*R*_Linkscale_ = 0.93, CI = 0.95, 1.00, *p* = <.0001). Examples can be seen in Figures 2, 5 and 7.
3. Coiled tubules – highly coiled or twisted, in some cases small, ‘blobby’ and unbranched, whilst in others they appeared longer and branched. Coiled tubules were uncommon, appearing in just 3 species (11.5%) (Figure 3). For each species that contained coiled tubules, they were common and found in both folds of the sample. In some cases, coiling was so extreme the tubule appeared ‘corkscrewed’ in shape. The categorisation of coiled tubules was highly repeatable between observers (*R*_Linkscale_ = 0.79, CI = 0.89, 1.00, *p* = .002). Examples can be seen in Figure 5.
4. Mini‐tubules – can be defined as being <30% the size of the largest tubule in the sample, and despite being very small relative to the longest tubules, often contained stored sperm. Due to the large number and consistency in length of mini‐tubules, we are confident that we were observing their full length in most cases. Occasionally, mini‐tubules appeared as barely longer than the tubule entrance. Mini‐tubules were very common, appearing to some degree in every species and in 50 folds (96.2% of fold samples) (Figure 3). Mini‐tubules could appear along the entire length of the sample but were particularly common towards the U region as the length of tubules generally declined closer to the uterus. Despite being small in length, the lumen and epithelial wall were similar in thickness to other tubules within the sample, and the tubule entrance was similar in size to other tubules in the sample. This distinguishes them from tubule‐like transitional tissue which are significantly smaller (e.g. Figure 4e). The categorisation of mini‐tubules was highly repeatable between observers (*R*_Linkscale_ = 0.99, CI = 0.98, 1.00, *p* = <.0001). Examples can be seen in Figure 5.
5. Agglomerate tubules – tubules were highly branched and appeared almost globular or star‐like in shape, sometimes appearing as dense clusters of tubules with multiple entrances. It was often unclear whether these clusters shared a lumen or were in fact distinct clusters of small agglomerate tubules. Transitional tissue may often appear agglomerate but has a less distinct form and appears only towards the U region of the sample (e.g. Figure 2c,d). Agglomerate tubules were found in 9 species (34.6% of species) and 15 folds (28.8% of fold samples) (Figure 3). There were 3 species in which agglomerate tubules were found in one fold of the sample but not the other (*Madagascar partridge*, mountain quail, and red legged partridge). The categorisation of agglomerate tubules was highly repeatable between observers (*R*_Linkscale_ = 0.93, CI = 0.95, 1.00, *p* = <.0001). Examples can be seen in Figures 5 and 7.

In addition to the 5 categories above, we noticed several samples contained tubules with interesting features that were not consistent or common enough to be considered separate categories, but are of note and worth discussing: first, we observed some tubules with unusually large/wide or ‘expanded’ entrances (Figure 6a) in helmeted guineafowl, Lady Amherst's pheasant and Reeve's pheasant (*Syrmaticus reevesii*); and second, we observed some tubules that were bulbous or inflated along their length (but had normal sized entrances) compared to the rest of the tubules in the C region of Chinese quail, Madagascar partridge and Swinhoe's pheasant samples (Figures 3 and 6b–d).

### 3.6 Categorisation flow chart

We created a flowchart for categorising tubule type based on our observations across species (Figure 7; and reproduced as a dichotomous key in the supplementary material). We found this process appropriate for categorising the tubules found in the species examined here, with the quality of images produced from our methodology, but recognise that there are likely to be more categories of tubules across other species/taxa and encourage researchers exploring tubule diversity to expand on our categories as necessary. Figure 3 provides a summary of the categories assigned to each species.

## 4 DISCUSSION

Avian SSTs are commonly described as ‘simple tubular invaginations’, located within a small strip of vagina tissue bordering the uterus, known as the UVJ (Bakst, 2011; Sasanami, 2017). They are widely assumed to look – and function in – the same way across all birds, according to the most frequently examined species: the domestic chicken, turkey, Japanese quail, and zebra finches. Here, we provide evidence that Galliformes – a large group of ground‐dwelling birds – exhibit striking and surprising variation in SST morphology across species. We propose the variety of tubules we observed across 26 species can be partitioned into 5 distinct categories: (1) Straight unbranched tubules; (2) straight branched tubules; (3) coiled tubules; (4) mini‐tubules; and (5) agglomerate tubules. We also show that above and beyond the apparent diversity in SST morphology, the surrounding tissue of the UVJ region appears to vary between species in some respects and includes at least 2 additional features: (1) channels and (2) transitional tissue, the function (if any) of which is currently unknown but warrants further investigation. Additionally, whether tubule function varies by category is unknown, but may have important consequences for our understanding of sperm storage and post‐copulatory sexual selection in general.

In most samples, tubules were found throughout the length of the UVJ. However, in half of all species, tubules did not always extend to the distal region of the sample that borders the uterus. The region directly preceding the uterus was often populated by what we refer to here as ‘transitional tissue’. Transitional tissue could not be defined as tubule tissue, surrounding vaginal tissue, or uterine tissue. It often appeared to contain smaller or poorly defined forms of the tubules seen further down the sample (Figure 4). While many of the structures observed in transitional tissue appeared to have entrances (when viewed under high magnification), due to their small size and the fact they were never observed to contain stored sperm, it seems unlikely they function as sperm storage structures. Previous work in yellow‐headed blackbirds (*Xanthocephalus xanthocephalus*) has shown that SSTs vary in length and likelihood to store sperm across regions of the UVJ (Briskie, 1996); it is possible that the transitional tissue observed here represents the smaller and less functional tubules found in that study. Another possibility is that transitional tissue represents a transitional/developmental state between the surrounding tissue and fully formed and functional storage structures. We currently do not know the processes of SST ontogeny, or fully understand how tubule morphology changes in response to the regression and subsequent regrowth of the oviduct in seasonally reproducing birds. Further work is needed to elucidate the precise ultrastructure and functional significance of transitional tissue.

Across the 26 Galliformes species examined here, we observed channel‐like depressions within the surrounding tissue of the UVJ in most samples. Channels varied in appearance between species, but were generally either very long, extending multiple fields of view, or were short and funnel‐like. Long channels (e.g. Figure 2b–d) often ended in a region populated by SSTs (e.g. Figure 2c), or in some cases ended directly in a tubule (Figure 2d). Shorter funnel‐like channels (e.g. Figure 2e,f) were most often observed at the start of the sample (i.e. the C end of the UVJ) and also frequently ended directly in a tubule.

It is difficult to discern the precise orientation of structures from a 2D image, and we cannot rule out the possibility that in some cases tubules may be stacked directly on top of channels rather than being connected; however, this seems unlikely to account for all observations, particularly for funnel‐like channels that were frequent and consistent in appearance. Further investigation is certainly warranted to confirm the presence and 3D ultrastructure of channels, and channel‐tubule connections, and to explore their functional significance. However, it seems likely that these channels are somehow involved in sperm transport or storage (at least to some degree). One convincing possibility is that channels simply provide a more direct route to the region of the vagina populated by tubules, or enhance swimming efficiency (Magdanz et al., 2015). The precise mechanism of sperm transport through the vagina is not known: in addition to sperm motility (Allen & Grigg, 1957), it has been suggested that some additional mechanisms may exist including ciliary movements of the surface epithelium of the vagina, contractions of the oviduct (possibly in combination with ciliary movement), and/or chemotactic guidance (Orr & Brennan, 2015; Sasanami, 2017). It is possible that in species that have them, channels aid in the rapid passage of sperm, and/or provide some protection from the environment of the vagina (e.g. the anti‐sperm immune response, vaginal sperm ejection, and mechanical flushes), while shorter funnel‐like channels may simply direct sperm into tubules once they reach the site of storage. Alternatively, close association with the vaginal mucosal epithelium might increase sperm contact with immune cells (Yoshimura et al., 1997), providing an opportunity for cryptic female choice to be intensified.

Sperm swimming mechanics are known to be strongly influenced by their interaction with surfaces (Denissenko et al., 2012). In mammals, sperm are thought to travel through micro‐channels in the reproductive tract, whereby motile sperm preferentially swim near the boundary of channel walls and this may act as a guiding mechanism towards the egg (Denissenko et al., 2012; Magdanz et al., 2015). Furthermore, there is some evidence that suggests that boundary‐following behaviour may be associated with higher sperm DNA integrity in humans (Eamer et al., 2016), and that channels may elicit intensified competition between sperm (Zaferani et al., 2019). Whether the channels we observe here are analogous to the micro‐channels of the mammalian female reproductive tract are unclear, but this possibility has potential implications for our understanding of sperm transport and post‐copulatory sexual selection within the avian vagina and warrants further investigation.

On the other hand, if tubules are directly connected to a channel, then sperm boundary following behaviour (Denissenko et al., 2012) may make it more difficult for sperm to exit storage without having to first travel back down towards the channel entrance. This would also be the case if long channels exist as enclosed tubes rather than open grooves (which is yet to be determined). This possibility raises the intriguing prospect that SSTs may not always function to store the fertilising subset of sperm, but instead may act as sperm ‘bins’ that inhibit – rather than facilitate – sperm participation in fertilisation. Sperm stored in SSTs are usually assumed to make up the fertilising subset of sperm because past studies in turkeys have shown that the number of sperm residing in SSTs is strongly correlated with the number that reach the ovum (Bakst, 2011; Brillard & Bakst, 1990). However, evidence that stored sperm always form the fertilising subset is lacking for other species, and so it may be worth revisiting this assumption. There is also evidence from other taxa that females can directly differentially remove sperm from storage, for example through differential ejection (aka sperm ‘dumping’), as seen in *Drosophila* (Snook & Hosken, 2004).

While the vagina is expected to be the major site of sperm selection in birds (Bakst, 2011; Orr & Brennan, 2015; Steele & Wishart, 1996), SSTs are now also predicted to play an important role in sperm selection by way of non‐random acceptance or release of sperm. For example, sperm filling rate into SSTs has been found to be unevenly distributed across the UVJ (Briskie, 1996; Ito et al., 2011; Sasanami et al., 2015); and in zebra finches, SSTs have been found to vary widely in diameter, possess constricted entrances that may act as a controlled barrier to sperm entry (Mendonca et al., 2019), and differentially store sperm from different inseminations (Hemmings & Birkhead, 2017). Sperm dumping and selective sperm displacement in other taxa (Barnett et al., 1995; Gasparini et al., 2018; Lüpold et al., 2012; Snook & Hosken, 2004) also supports the role of storage structures in sperm selection. In bats, species that store sperm also appear to produce more sperm, and testis size is correlated with sperm storage duration suggesting that longer sperm storage is associated with increased sperm competition (Orr & Brennan, 2015; Orr & Zuk, 2013). The large variation in SST morphology observed in the current study may lend support to the hypothesis that SSTs exhibit functional differences that could influence the outcome of sperm competition. In accordance with this, we also observed a non‐uniform distribution of stored sperm among tubules, suggesting individual SSTs may vary in their ability to accept, store and release sperm. Sperm were also commonly observed distributed along the entire length of the tubules, rather than being congregated just within the blind‐end; however, we did not notice any patterns in sperm distribution across species or SST category.

Theoretically, tubule shape and length may influence the speed or order in which sperm are released from storage. Briskie (1996) found that in yellow‐headed blackbirds, uterine‐end SSTs were smaller and later to mature but released a greater number of sperm during the egg‐laying period, suggesting that smaller tubules may accept sperm later, but release them more quickly, than longer tubules. Accordingly, sperm stored in the shorter mini‐tubules we observe here (e.g. Figure 4g), may exit storage more quickly than sperm stored at the end of longer or more highly branched tubules (e.g., Figure 5c). Branch length and distance from the SST entrance will presumably influence how easily/fast sperm are released, potentially providing a mechanism of control over the order of sperm release from storage. Variation in tubule shape within samples may therefore also help to reduce mating order effects, by creating variation in ‘the playing field’, in which sperm from different males are stored in a variety of tubules of varying length, branch number, and complexity, and with a varying advantage/disadvantage over the timing of release (Hemmings & Birkhead, 2017).

We observed that agglomerate tubules are often extremely highly branched, complex, and diverse in morphology (e.g. Figure 5i,j). How this structural diversity might translate to variation in SST function is unclear and introduces several questions. For example, do all branches function in the same way or are there selective features that allow branches to vary in their ability to accept sperm? Does mating order influence which branch sperm are accepted into? Do the number of branches of a tubule influence the ease with which sperm can exit the tubule upon release? Given the degree of variation in SST structures observed here, and the highly diverse nature of sperm cells (Pitnick et al., 2009), a logical line of questioning would be to assess whether SST morphology and complexity correlate with sperm morphology, sperm storage duration, and/or sexual selection intensity (which is typically correlated with mating system) across species. One possibility is that highly branched or complex tubule structures introduce variation in the ease with which sperm can enter or reach the most protective regions of the storage tubule. For example, if more vigorous sperm are better able to reach more distal branches, they may be better protected from further selective processes such as sperm ejection or the anti‐sperm response within the vagina. Exploring differential storage of sperm of varying quality across a variety of tubule types may shed light on whether tubule morphology is linked to sperm selection.

In the case of coiled tubules (e.g., Figure 5d–f), the tightness of coils or their length may provide an even more extreme degree of manipulation over the ability of sperm to enter storage, or the timing and speed of sperm release. The structure of coiled tubules has some interesting parallels with the coiled vaginas of some species of waterfowl, where the coiled shape of the reproductive tract has a sexually antagonistic co‐evolutionary relationship with the coiled penis of the male (which is coiled in the opposite direction to the vagina, making intromission more difficult) (Brennan et al., 2010). In addition to their spiral structure, the vaginas of these species also have multiple blind‐ended ‘pouches’, in which the penis can be directed to act as a mechanism of female control over sperm use following forced copulations (Brennan et al., 2010). Figure 2f presents a striking example of a tubule in which one branch (notably, the branch closest to the tubule entrance) is coiled whilst the other is not. One possibility is that coiled tubules function in a similar way to the blind‐ended and spiral‐shaped vaginas of ducks, possibly providing a sperm ‘bin’ by which a proportion of sperm are prevented from being released, or released quickly enough, to participate in sperm competition.

Another comparison can be drawn with the uterine muscular coiling (UMC) of viperid snakes. UMC involves a contraction of the innermost layers of the UVJ, which causes the tissue to form a coiled shape (not dissimilar to the coiled appearance of tubules seen in Figure 5d–f) (Muniz‐Da‐Silva et al., 2020). It is thought that UMC may function as a mechanism of sperm storage by maintaining the position and viability of sperm. There is evidence that SSTs in turkeys are innervated, and individual SSTs house F‐actin rich terminal webs in the epithelium that exhibit a coiled appearance, suggesting they are capable of contraction (Freedman et al., 2001). Nerve fibres have also been observed in close association with SSTs in the alpine accentor (*Prunella collaris*) (Chiba & Nakamura, 2001), although contractile elements were not found in this case. In support of the contractile potential of SSTs, a contraction‐like change to SST morphology was observed in Japanese quail following an injection of progesterone (which is thought to be one factor that triggers sperm release from tubules) (Ito et al., 2011), suggesting that individual SSTs are capable of contraction and relaxation under presumably fine temporal control. It may therefore be possible that tubule coiling is a plastic mechanism (under neural or hormonal control) for maintaining and protecting sperm in storage. Interestingly, it is reported that UMC in snakes becomes less visible in tissue preserved in 10% formalin relative to fresh tissue. It may therefore be worth examining the morphology of SSTs across birds using fresh, rather than preserved tissue, and across different stages of reproduction (e.g., before, during, and after sperm acceptance and release). Exploring SST morphology in fresh tissues will ensure that any features lost as a result of the preservation process are uncovered. Coiled tubules have in fact been observed at least once before, in a study examining the morphology of SSTs in the American kestrel (*Falco sparverius*) (Bakst & Bird, 1987). Their shape was only alluded to briefly and their significance was not discussed at the time nor (to our knowledge) since. We believe our images provide the first evidence for such structures in Galliformes. While we have hypothesised several possible functional explanations for coiled SSTs, further work is needed to uncover the significance of these structures for our understanding of SST function and post‐copulatory sperm storage and selection in general.

Finally, we observed two additional features that were uncommon and inconsistent within samples, suggesting they were not appropriate for inclusion as separate categories. (1) Expanded entrances – these unusual tubules were observed to have abnormally wide and funnel‐like entrances. SSTs have been shown to have constricted gate‐like entrances (Mendonca et al., 2019), and so it could be that these tubules were simply in the processes of relaxing following sperm release or prior to sperm acceptance. Future work exploring SST morphological changes through time is a challenging but necessary step to understanding the process of sperm acceptance and release. (2) Bulbous tubules – whether these are typical structures for these species is unclear, but these tubules were seen containing stored sperm and are clearly functional. Further work exploring intraspecific variation in tubule morphology will be needed to explore this. If these structures do appear to be consistent features within and across species, re‐evaluation of their inclusion as a separate tubule category may be needed.

## 5 CONCLUSIONS

SSTs in birds are often described as ‘simple tubular invaginations’. Whilst that may be true of the tubules typically seen in well studied birds like chickens and turkeys, we find that across other Galliformes, tubule structure is far more variable, diverse, and complex than previous assumed. The variation in tubule and surrounding tissue structure we observe here may have important implications for our understanding of sperm storage tubule function in general, with broader consequences for our understanding of post‐copulatory sperm selection. Further research is needed to quantify this variation across Galliformes, explore variation across other species of birds, and determine the functional significance of the structures we observe here. An obvious first step will be to explore whether storage tubule types are associated with sperm morphology, sperm storage capacity and sperm competition intensity. It may also be useful for future work to examine different sperm storage types using scanning and transmission electron microscopy, including the use of 3D imaging techniques, which will help confirm their ultrastructure, internal morphology, and relationships with surrounding tissues. Ultimately, these findings contribute to the growing body of evidence across taxa that female sperm storage structures can be complex, highly specialised and variable (Beese & Baur, 2006; Berger et al., 2011; Holt & Fazeli, 2016; Hopkins et al., 2020; Lüpold et al., 2013; Orr & Brennan, 2015; Ward, 2000).

## AUTHOR CONTRIBUTIONS

**Katherine Assersohn:** Conceptualization (equal); data curation (lead); formal analysis (lead); investigation (lead); methodology (equal); project administration (equal); software (lead); validation (lead); visualization (lead); writing – original draft (lead); writing – review and editing (equal). **J. Paul Richards:** Conceptualization (supporting); methodology (equal); writing – review and editing (supporting). **Nicola Hemmings:** Conceptualization (equal); data curation (supporting); funding acquisition (lead); investigation (supporting); methodology (equal); project administration (equal); resources (lead); supervision (lead); validation (supporting); writing – review and editing (equal).

## FUNDING INFORMATION

KA was supported by the Natural Environment Research Council ACCE Doctoral Training Part‐nership (DTP) (grant number NE/S00713X/1). NH was supported by a Royal Society Dorothy Hodgkin Research Fellowship (DHF160200) and a University of Sheffield Woman Academic Returners' Programme (WARP) award which funded JPR's position.

## CONFLICT OF INTEREST STATEMENT

The authors declare there are no conflicts of interest.

## Supporting information


Appendix S1.

## Data Availability

No quantitative data were generated.
