# Supplementary material for: The surprising complexity and diversity of sperm storage structures across Galliformes
Source: Ecol Evol. 2024 Jun 21;14(6):e11585. doi: 10.1002/ece3.11585 (PMC11190584; doi:10.1002/ece3.11585)
Supplement: Supplementary file 1 — Appendix S1. [file ECE3-14-e11585-s001.docx]

**Dichotomous key for determining sperm storage tubule morphology according to the categories presented in ‘The surprising complexity and diversity of sperm storage structures across Galliformes’ (Assersohn et al., 2024).**

**1a** The storage structures are clear and well-defined (located in the vagina, distinct and markedly different from the surrounding tissue) ***Go to step 2***

**1b** The storage structures are not clear or well-defined (structures are not markedly different from the surrounding tissue) ***Go to step 3***

**2a** Structures are very small compared to the largest tubule in the sample (tubules are <30% the size of the largest tubules in the sample) ***Go to step 4***

**2b** Structures are **not** very small compared to the largest tubule in the sample (tubules are at least 30% the size of the largest tubules in the sample) ***Go to step 6***

***3a*** Structures appear towards the uterus end of the sample (in the vagina, but between the population of ‘normal’ tubules and the uterus) ***Go to step 5***

***3b*** Structures do not appear in the uterus end of the vagina *–* ***UNDEFINED STRUCTURE.***

*Structures may fall outside of this categorisation key for the following reasons:*

- *Structure is not storage or storage-related tissue*
- *Image quality is not sufficient to observe structures*
- *The structure is currently undescribed*

**4a** Structures look like the other tubules in the sample in all but length. They have similar sized entrances, epithelium wall and lumen diameter to ‘normal’ tubules, but are just much shorter in length *-* ***MINI-TUBULES***

**4b** Structures differ from the other tubules, they are much shorter in length but also have different sized entrances, epithelium walls, and lumen diameter. ***Go to step 3***

**5a** Structures appear as densely concentrated, but very small tubule-like structures. They are much smaller than mini-tubules, and as they become denser, they transition into uterus tissue – ***TRANSITIONAL TISSUE***

**5b** Structures do not appear like densely concentrated, but very small tubule-like structures. They are not smaller than mini-tubules and do not appear to transition into uterus tissue. ***Go to step 7***

**6a** Tubules are simple ‘straight’ tubes. They may be curved, but not coiled/twisted, globular or agglomerate. ***Go to step 8***

**6b** Tubules are not simple ‘straight’ tubes. They may be coiled/twisted, globular or agglomerate. ***Go to step 9***

**7a** Structures appear ‘fluffy’ or ‘cloud-like’. They look like poorly defined agglomerate tubules, but are distinct from uterus tissue. They may transition from agglomerate tubules into uterus tissue – ***TRANSITIONAL TISSUE***

**7b** Structures do not appear ‘fluffy’ or ‘cloud-like’ **­*–*** ***UNDEFINED STRUCTURE***

*Structures may fall outside of this categorisation key for the following reasons:*

- *Structure is not storage or storage-related tissue*
- *Image quality is not sufficient to observe structures*
- *The structure is currently undescribed*

**8a** Structures are ‘channel-like’ in appearance. They may begin at the cloaca-end of the vagina, or further up the UVJ. They may span more than 1 field of view, and lead into tubule tissue. Some may terminate directly into a tubule ***­– CHANNEL TISSUE***

**8b** Structures are not ‘channel-like’ in appearance. ***Go to step 10***

**9a** Structures are agglomerate in appearance – they are highly branched or very dense clusters of indistinguishable tubules. Structure appears somewhat globular. Branches may appear from a larger central ‘body’.  ***– AGGLOMERATE***

**9b** Structures are not agglomerate in appearance. They do not appear as highly branched or very dense clusters of indistinguishable tubules. ***Go to step 11***

**10a** Structures are branched. They are clear tubules with an obvious split into a clear branch i.e., they are not just overlaying tubules, which will have >1 lumen. ***– STRAIGHT BRANCHED***

**10b** Structures are not branched. They are clear, single branched blind-ended tubes with no splits and only one lumen. ***– STRAIGHT UNBRANCHED***

**11a** Structures are twisted/coiled. They are distinct separate tubules, but they appear coiled or twister. They may be branched or unbranched ***- COILED***

**11b** Structures are not coiled or twisted. **­*–*** ***UNDEFINED STRUCTURE***

*Structures may fall outside of this categorisation key for the following reasons:*

- *Structure is not storage or storage-related tissue*
- *Image quality is not sufficient to observe structures*
- *The structure is currently undescribed*
